# A Comparative Study on CT-guided Radiofrequency Ablation and Targeted Therapy: Intervention Efficacy and Survival Rates in Lung Cancer Patients

**DOI:** 10.2174/0115734056311827241211092432

**Published:** 2025-01-02

**Authors:** Tianyu Zhao, Chunjing Zhang, Hang Dai, Jingyu Li, Liguo Hao, Yanan Liu

**Affiliations:** 1 Medical Technology Department, Qiqihar Medical University, Qiqihar 161006, Heilongjiang, China; 2 Foreign Language Department, Qiqihar Medical University, Qiqihar 161006, Heilongjiang, China

**Keywords:** Lung cancer, Radiofrequency ablation, Targeted therapy, Clinical efficacy, Immune response, Safety, Survival time

## Abstract

**Objective:**

The study aimed to evaluate the clinical efficacy of CT-guided radiofrequency ablation in conjunction with targeted therapy in lung cancer patients.

**Methods:**

We retrospectively analyzed 80 lung cancer patients. They were stratified into the Observation Group (OG; n=40, treated with CT-guided radiofrequency ablation in conjunction with targeted therapy) and the Control Group (CG; n=40, treated solely with targeted therapy).

**Results:**

The Overall Response Rate (ORR) and Disease Control Rate (DCR) in the OG group (70.00%, 95.00%) were significantly higher than those in the CG group (57.50%, 87.50%). After 6 weeks of treatment, the OG group had significantly lower levels of SCC, CEA, and CA125, higher CD4+ levels, and lower CD8+ levels, compared to the CG group. The 24-month follow-up survival rate of the OG group (47.50%) was significantly higher than that of the CG group (27.50%).

**Conclusion:**

CT-guided radiofrequency ablation and targeted therapy have been proven effective in treating lung cancer.

## 1 INTRODUCTION

According to the “2020 Global Cancer Burden Update”, global cancer-related mortality has surpassed 9.96 million. Among these cases, lung cancer is the most lethal, accounting for 1.8 million deaths, thus surpassing all other forms of cancer and ranking first in terms of cancer-related mortality [1]. The National Tumor Registry Center reported that in 2014, China's lung cancer incidence and mortality rates were 57.13/100,000 and 45.80/100,000, respectively, placing lung cancer at the top of the list for malignant tumors. In 2020, cancer claimed 3 million lives in China, with lung cancer contributing to 710,000 deaths, comprising 23.8% of the total cancer deaths and remaining at the forefront of mortality rates [2]. Globally, lung cancer is now the most significant oncological threat to human health [3].

Lung cancer's insidious clinical manifestations during its initial stages mean that most patients are only diagnosed at an advanced stage. While radiotherapy and surgery have been implemented as clinical interventions, their effectiveness has been contested [4, 5].

In recent years, innovative measures for treating lung cancer have been under exploration, such as targeted drug intervention, combined radiotherapy and chemotherapy, gene regulation, *etc*. These measures have shown promise in enhancing the long-term survival rate of lung cancer patients to a certain degree. The combination of CT-guided radio-frequency ablation and conventional chemotherapy has proven effective in treating lung cancer. A follow-up study involving 90 lung cancer patients discovered that compared to those treated with conventional surgery, patients undergoing CT-guided radiofrequency ablation demonstrated significantly higher remission rates. Additionally, short- and long-term follow-ups showed a superior survival rate and improved quality-of-life scores, thereby affirming the utility of this therapeutic modality [6].

Presently, there is a dearth of research on the combined application of CT-guided radiofrequency ablation and targeted drugs in lung cancer patients. In this paper, our retrospective research ascertains that the combination of CT-guided radiofrequency ablation and targeted drugs offers superior therapeutic efficacy and survival rates for lung cancer patients compared to conventional targeted drugs alone. A detailed account of this investigation is provided below.

## 2 MATERIAL AND METHODS

### 2.1 Study Design and Patient Selection

A retrospective design was employed to collect the clinical data of 80 lung cancer patients treated in our hospital between August 2018 and May 2021. The data were garnered from a review of electronic medical records. The inclusion and exclusion criteria are as follows:

#### 2.1.1 Inclusion Criteria

(1) Pathologically and radiologically confirmed lung cancer diagnosis; (2) clinical staging (TNM version 8) stage III or IV; (3) aged 18 years or above; (4) Eastern Cooperative Oncology Group (ECOG) physical status score [7] between 0 and 2; (5) as per the World Health Organization immune-based Response Evaluation Criteria in Solid Tumors (iRECIST) [8], the presence of one or more lesions that permit precise radial line measurement; (6) availability of comprehensive clinical data; (7) patients deemed clinically unsuitable for surgical treatment, with reference to the “Expert Consensus on Image-guided Radiofrequency Ablation of Pulmonary Tumors (2018 version)”, recommended to undergo radiofrequency ablation or other treatment measures. This study was approved by the ethics committee of Qiqihar Medical University [no.: (2021)85].

#### 2.1.2 Exclusion Criteria

(1) Concurrent autoimmune diseases (such as rheumatoid arthritis, systemic lupus erythematosus, *etc*.); (2) concomitant serious chronic diseases (including AIDS, hepatitis B, hepatitis C, *etc*.); (3) concurrent severe organ failure (like heart failure, renal failure, *etc*.); (4) uncontrollable diabetes mellitus or hypertension; (5) pregnancy or lactation; (6) concurrent psychiatric disorders.

### 2.2 Data Collection

**Fig. (1):**
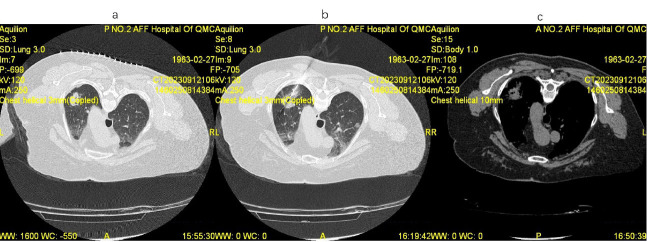
Preoperative, intraoperative, and postoperative imaging of a typical patient. (**a**): preoperative imaging; (**b**): intraoperative imaging; (**c**): postoperative imaging.

Utilizing our in-house medical system, baseline clinical data, encompassing age, sex (SAGER guidelines were followed), smoking status, cancer type (small-cell lung cancer, lung adenocarcinoma, lung squamous carcinoma, or large-cell lung cancer), clinical stage (stage III or IV), tumor location (left lung, right lung, or bilateral), tumor diameter, ECOG scores, tumor markers [levels of Squamous Cell Carcinoma (SCC) antigen, Carcinoembryonic Antigen (CEA), and Carbohydrate Antigen 125 (CA125) upon admission and post-six weeks of treatment], immune indices (CD4+, CD8+, recorded upon admission and post six weeks of treatment), preoperative, intraoperative, and postoperative imaging of the patient (Fig. **1**), the occurrence of adverse reactions (recorded either from telephonic follow-up or during follow-up visits within the electronic system), and short-term and long-term outcomes, were obtained.

Short-term outcomes refer to treatment results assessed six weeks post-intervention, classified into Complete Response (CR), Partial Response (PR), Stable Disease (SD), and Progressive Disease (PD) per iRECIST, wherein Overall Response Rate (ORR) = CR + PR and Disease Control Rate (DCR) = CR + PR + SD. Long-term outcomes represented patient status as of May 2023, recorded according to the last patient tracked for 24 months, to document patient survival and compute the patient survival rate.

Data were collected by the investigator in collaboration with the patient's assigned physician, with data collection commenced in August 2018 and concluded in May 2021.

### 2.3 Outcome Measures

Following data collection and screening, we identified 40 patients diagnosed with lung cancer who underwent CT-guided radiofrequency ablation combined with targeted therapy (utilizing gefitinib, cisplatin, and paclitaxel) in our hospital from August 2018 to May 2021 (designated as the OG group). We compared these to 40 patients who received targeted therapy alone (gefitinib, cisplatin, and paclitaxel) during the same timeframe (termed the CG group).

### 2.4 Statistical Analysis

We utilized SPSS 22.0 for data processing. The metric data are presented as mean ± standard deviation and analyzed using t-tests, while categorical data are expressed as rates and analyzed with chi-square tests. Kaplan-Meier curves were generated to illustrate patients' postoperative survival trajec-tories. A *p*-value of less than 0.05 was considered statistically significant.

## 3 RESULTS

### 3.1 Comparison of Baseline Clinical Data between Patient Groups

Baseline clinical parameters, including sex, age, smoking status, cancer type, clinical staging, tumor location, tumor diameter, and others, showed no significant differences between groups (*p*>0.05), suggesting suitable comparability (Table **1**).

### 3.2 Comparative Analysis of Recent Therapeutic Efficacy

**Fig. (2):**
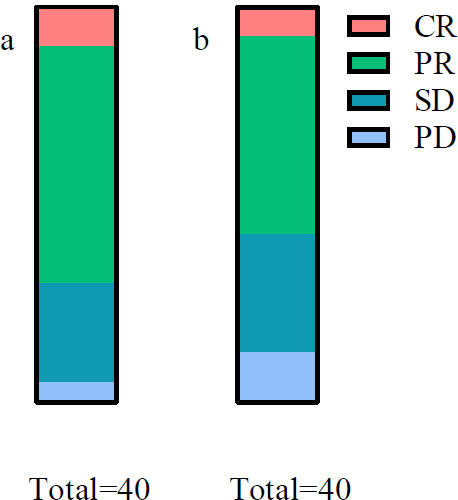
Comparison of recent efficacy of treatment. (**a**): observation group; (**b**): control group. **Note: **CR: complete response; PR: partial response; SD: stable disease; PD: progressive disease.

In the OG group, we observed 4 cases of CR, 24 cases of PR, 10 cases of SD, and 2 cases of PD, with an ORR of 70.00% (28/40) and a DCR of 95.00% (38/40). The CG group comprised 3 cases of CR, 20 cases of PR, 12 cases of SD, and 5 cases of PD, with an ORR of 57.50% (23/40) and a DCR of 87.50% (35/40). Both the ORR and DCR were significantly higher in the OG group (*p*<0.05) (Table **2** and Fig. **2**).

### 3.3 Variations in Tumor Marker Levels Pre- and Post-treatment

**Fig. (3):**
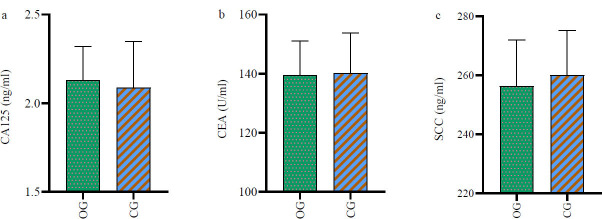
Difference in tumor marker levels between the two groups of patients before treatment. There was no statistically significant difference between the two groups in the levels of tumor markers CA125 (**a**), CEA, and SCC (**b**) between the two groups (*p*>0.05). **Note: **OG: observation group; CG: control group; SCC: squamous cell carcinoma antigen; CEA: carcinoembryonic antigen; CA125: carbohydrate antigen 125.

**Fig. (4):**
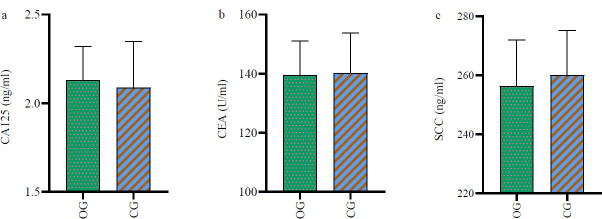
Difference in tumor marker levels between the two groups after 6 weeks of treatment. The levels of CA125 (Fig. **3a**), CEA (Fig. **3b**), and SCC (Fig. **3c**) were lower in the OG group than in the control group after 6 weeks of treatment (*p*<0.05). * *p*<0.05. **Note: **OG: observation group; CG: control group; SCC: squamous cell carcinoma antigen; CEA: carcinoembryonic antigen; CA125: carbohydrate antigen 125.

No significant intergroup differences in the pre-treatment levels of SCC, CEA, and CA125 were observed (*p*>0.05). However, the OG group exhibited significantly lower levels of these markers six weeks post-treatment (*p*<0.05) (Table **3**, Figs. **3** and **4**).

### 3.4 Changes in Immune Indices Pre- and Post-treatment

**Fig. (5):**
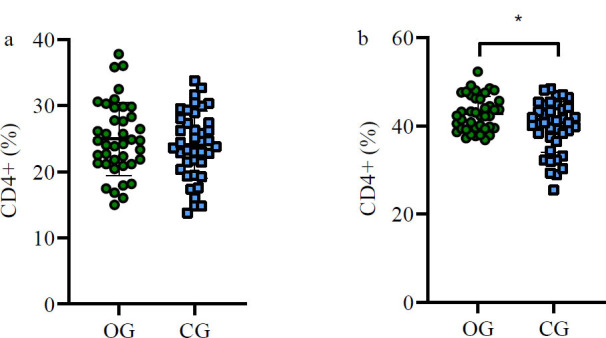
Variation in CD4+ levels pre- and post-treatment. (**a**) Prior to treatment, the discrepancy in CD4+ levels between both groups was not statistically significant (**b**). However, following a six-week treatment period, patients in the OG group exhibited elevated CD4+ levels relative to their CG group counterparts (*p*<0.05). Note: *p<0.05. OG: observation group; CG: control group.

**Fig. (6):**
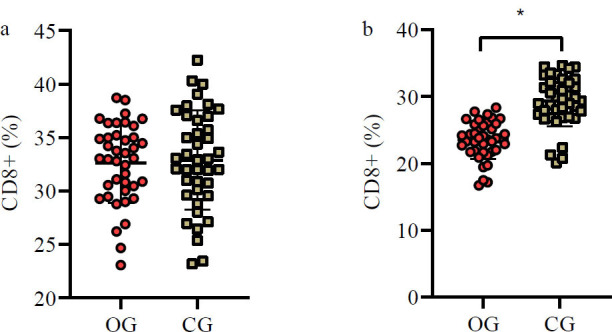
Fluctuation in CD8+ levels pre- and post-treatment. (**a**) Similar to CD4+ levels, no significant difference was found in CD8+ levels between the groups prior to treatment. (**b**). Conversely, after six weeks of treatment, the OG group demonstrated significantly diminished CD8+ levels compared to the CG group (*p*<0.05). Note: *p<0.05. OG: observation group; CG: control group.

No significant differences were found in CD4+ and CD8+ levels across groups before treatment (*p*>0.05). However, six weeks post-treatment, the OG group showed significantly higher CD4+ and lower CD8+ levels compared to the CG group (*p*<0.05) (Table **4**, Figs. **5** and **6**).

### 3.5 Comparison of Adverse Reaction Incidences

**Fig. (7):**
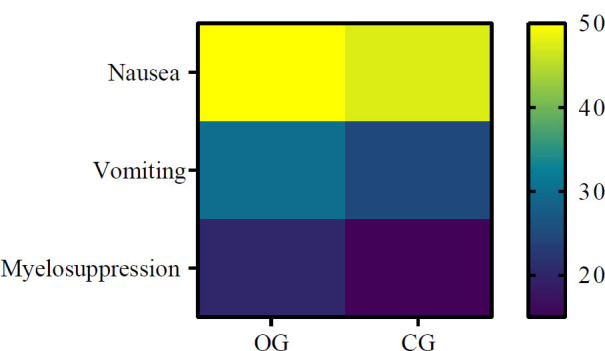
Incidence of adverse reactions in both groups. The prevalence of adverse reactions, including nausea, vomiting, and myelosuppression, did not demonstrate a statistically significant difference between the OG and CG groups. **Note: **OG: observation group; CG: control group.

In the OG group, 20 patients experienced nausea, 12 had vomiting episodes, and 8 reported myelosuppression. The CG group included 19 cases of nausea, 10 of vomiting, and 6 of myelosuppression. These differences were not statistically significant (*p*>0.05) (Fig. **7**).

### 3.6 Comparative Analysis of Long-term Follow-up Efficacy

**Fig. (8):**
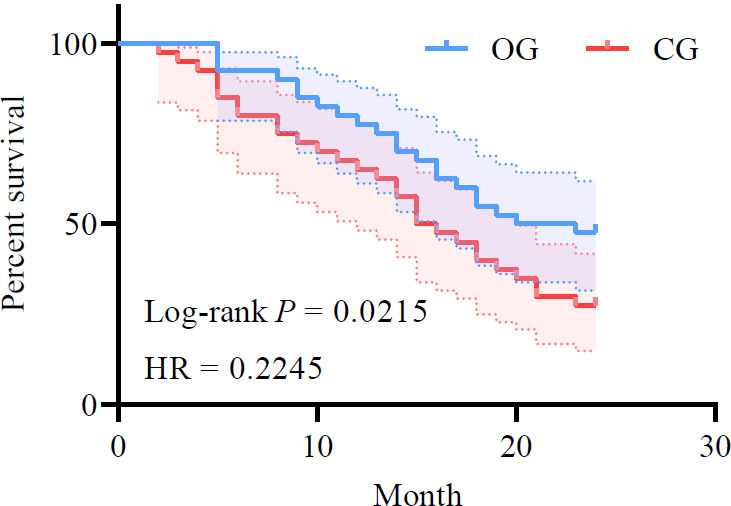
Comparison of survival rate between two groups. **Note: **OG: observation group; CG: control group.

After 24 months of follow-up, the OG group showed a higher survival rate (47.50%, 19/40) compared to the CG group (27.50%, 11/40) (*p*<0.05). The Progression-free Survival (PFS) duration of the OG group (9.68±1.32 months) was also greater than that of the CG group (8.69±0.59 months) (*p*<0.05) (Fig. **8**).

## 4 DISCUSSION

Lung cancer, the most prevalent and deadly malignant tumor globally, poses a grave threat to human health and safety. As indicated by data from the National Tumor Registry Center in 2017, China's lung cancer incidence and mortality rates in 2014 stood at 57.13/100,000 and 45.80/100,000, respectively, ranking it as the deadliest malignancy [9]. Information provided by the World Health Organization (WHO) asserts that lung cancer is the leading cause of cancer-related mortality in men and the second leading cause in women [10]. Factors, such as the aging population, environmental pollution, and accelerated lifestyles, have led to a steady increase in lung cancer prevalence in China, with the country now housing the world's highest number of lung cancer patients [11]. Although surgical interventions, radiotherapy, and targeted therapy can mitigate the mortality rate of patients with advanced lung cancer to a certain degree, a significant proportion of patients do not experience symptom relief or may even undergo disease progression post-standard first or second-line treatments [12]. Molecular targeted drugs research emphasizes that while these drugs effectively ameliorate clinical symptoms of advanced lung cancer patients, they can provoke drug resistance upon prolonged use, necessitating the urgent search for combinatorial therapeutic approaches to compensate for such deficits [13, 14].

The present study analyzed the clinical efficacy of augmenting targeted therapy with CT-guided radiofrequency ablation in lung cancer patients by comparing the outcomes with a control group. Findings indicated that patients in the OG group, who received the combined therapy, showed substantial superiority in terms of near-term therapeutic efficacy relative to the CG group receiving targeted drugs alone, as demonstrated by a higher ORR (70.00% *vs*. 57.50%) and a DCR (95.00% *vs* 87.50%). These results were found to be in line with Chockalingam *et al.*'s findings [15]. Chockalingam *et al.* concluded through a meta-analysis that percutaneous ablation is gaining favor over traditional surgical resection in some advanced lung cancer patients. They suggested that integrating radiofrequency ablation with traditional treatments might herald a new trend in lung cancer treatment. Our analysis indicated that radiofrequency ablation, a method that employs electromagnetic waves to directly target and eradicate cancer cells, causes rapid tumor cell dehydration, protein denaturation, and DNA strand breakage at high temperatures (90-110°C). Additionally, the elevated temperature coagulates blood vessels surrounding cancer cells, thereby blocking cancer cell metastatic pathways [16, 17]. Targeted therapy drugs, on the other hand, directly target specific molecules to inhibit cancer cell growth, proliferation, and metastasis [18, 19]. Hence, CT-guided radiofrequency ablation conjoined with targeted therapy presents a dual strategy, exterminating cancer cells and obstructing their proliferation and metastasis simultaneously, thus resulting in enhanced therapeutic effects.

This study also compared the differences in tumor markers as well as immune indicators between the OG and CG groups before and after treatment. The results revealed that the OG group exhibited significantly lower levels of tumor markers post-treatment, a pattern consistent with findings obtained by Wei *et al.* [20]. Wei *et al.* demonstrated that the therapeutic intervention combining karelizumab and microwave ablation significantly reduced multiple inflammatory factors (*e.g*., IL-6, TNF-α) and tumor marker levels (CEA, SCC, *etc*.) in non-small cell lung cancer patients, a more substantial decrease than in patients undergoing medication-only treatment. It was analyzed in this paper that lung cancer patients often expe-rience significant immune function suppression due to immune responses, particularly noticeable during chemotherapy. Factors, such as CD4+ and CD8+ levels, instrumental in the specific cellular immune response, tend to correlate with a patient's immune function. In this study, patients in the OG group demonstrated higher levels of CD4+ and lower levels of CD8+ post-treatment, compared to the CG group. Moreover, tumor markers, such as SCC and CEA, were lower in the OG group. These findings validated that CT-guided radiofrequency ablation combined with targeted therapy can aid in regulating lung cancer patients' immune function, playing a pivotal role in improving long-term prognosis, as corroborated by studies conducted by Miller *et al.* [21] and Hattori *et al.* [22]. Ultimately, survival rate comparisons between the two patient groups further validated the hypothesis that modulating immune function in lung cancer patients can ameliorate prognosis. Therefore, CT-guided radiofrequency ablation combined with targeted therapy exhibits considerable significance for widespread application and dissemination.

Although this study retrospectively analyzed the application value of CT-guided radiofrequency ablation combined with targeted therapy in lung cancer patients and obtained detailed data, it should also be recognized that there were many shortcomings in this study. For example, the retrospective analysis of the data in this study may have been affected by overlooked biases and confounding factors. Although completeness of the available patient data was required, the enrolled patients were from a single source representing a small sample size. A prospective, large-sample, and multicenter randomized controlled study will be conducted in the future to help avoid the impact of the above situation on the research results.

## CONCLUSION

CT-guided radiofrequency ablation combined with targeted therapy demonstrated effectiveness in lung cancer treatment, enhancing near- and long-term clinical outcomes, prolonging patient survival, improving the inflammatory state, and offering confirmed safety.

## Figures and Tables

**Table 1:** Comparison of differences in baseline clinical data between the two groups [n (%), 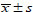].

General Clinical Information		OG Group (n=40)	CG Group (n=40)	*t/χ^2^*	*P*
Sex	Men	30	31	0.008	0.962
	Women	10	9		
Average age (years)		60.23±5.16	59.86±5.81	1.235	0.115
Smoking	Yes	23	21	0.016	0.815
	No	17	19		
Type	Small cell lung cancer	30	26	0.126	0.812
	Lung adenocarcinoma	6	8		
	Lung squamous carcinoma	3	4		
	Large cell carcinoma	1	2		
Clinical stage	III	26	29	0.035	0.815
	IV	14	11		
Tumor site	Left lung	16	18	1.331	0.512
	Right lung	18	15		
	Double lung	6	7		
Tumor Diameter	<3.0cm	6	5	0.169	0.881
	3.0-4.0cm	26	28		
	>4.0cm	8	7		
ECOG score	0-1 point	26	27	0.062	0.789
	2 points	14	13		

**Table 2:** Comparison of recent therapeutic efficacy [n (%)].

Group	Cases	CR	PR	SD	PD	ORR	DCR
OG group	40	4	24	10	2	28 (70.00)	38 (95.00)
CG group	40	3	20	12	5	23 (57.50)	35 (87.50)
χ^2^	-	-	-	-	-	4.265	3.265
P	-	-	-	-	-	0.015	0.039

**Table 3:** Differences in tumor marker levels before and after treatment in the two groups (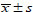).

Group	Cases	SCC (ng/ml)		CEA (U/ml)		CA125 (ng/ml)	
		Before Treatment	After 6 Weeks of Treatment	Before Treatment	After 6 Weeks of Treatment	Before Treatment	6 Weeks After Treatment
OG group	40	2.13±0.19	1.32±0.51*a*	139.56±11.56	112.15±16.33 a	256.32±15.69	213.01±16.56 *a*
CG group	40	2.09±0.26	1.86±0.26 *a*	140.15±13.65	129.56±15.71 *a*	260.15±15.11	232.56±18.11 *a*
t	-	0.531	15.127	0.653	5.163	0.418	6.536
P	-	0.663	0.000	0.441	0.001	0.369	0.000

**Note: **Compared to the same group before treatment, a *p*<0.05.

**Table 4:** Differences in immune indexes before and after treatment in the two groups of patients.

Group	Cases	CD4+ (%)		CD8+ (%)	
		Before Treatment	After 6 Weeks of Treatment	Before Treatment	After 6 Weeks of Treatment
OG group	40	25.62±5.16	44.19±5.32 *a*	32.15±4.16	23.15±2.98 *a*
CG group	40	24.98±5.98	38.98±5.07 *a*	31.98±4.98	28.96±3.65 *a*
t	-	0.663	5.116	0.771	6.983
P	-	0.418	0.000	0.236	0.000

**Note: **Compared to the same group before treatment, a represents *p*<0.05.

## Data Availability

The data and supportive information are available within the article.

## LIST OF ABBREVIATIONS

ECOG: Eastern Cooperative Oncology Group
iRECIST: immune-based Response Evaluation Criteria in Solid Tumors
SCC: Squamous Cell Carcinoma
CEA: Carcinoembryonic Antigen
CR: Complete Respons
PR: Partial Response
SD: Stable Disease

## STANDARDS OF REPORTING

STROBE guidelines were followed.
